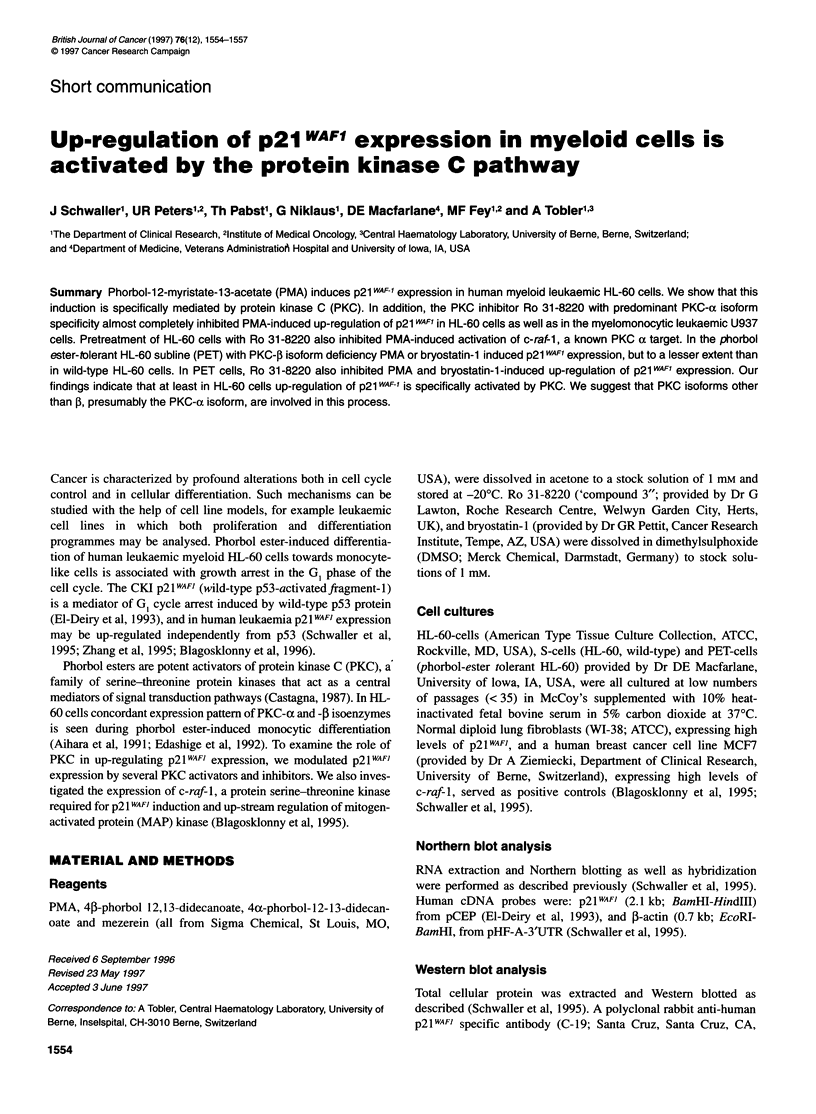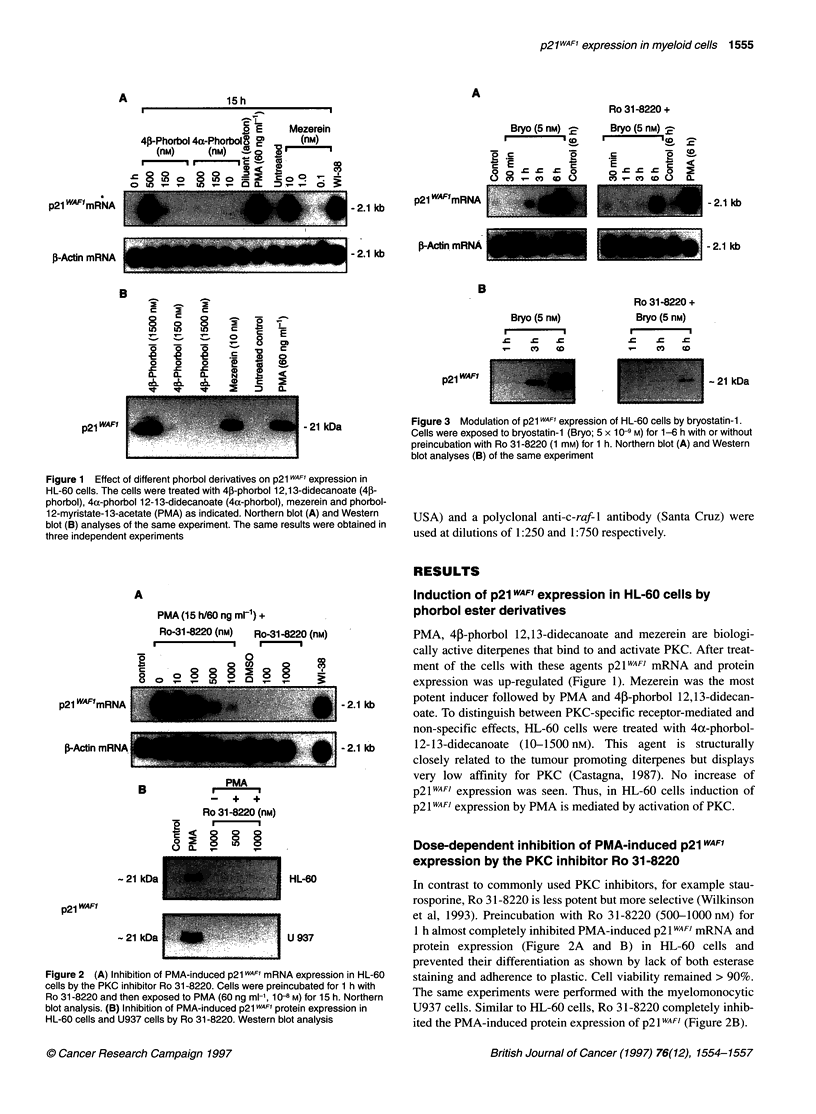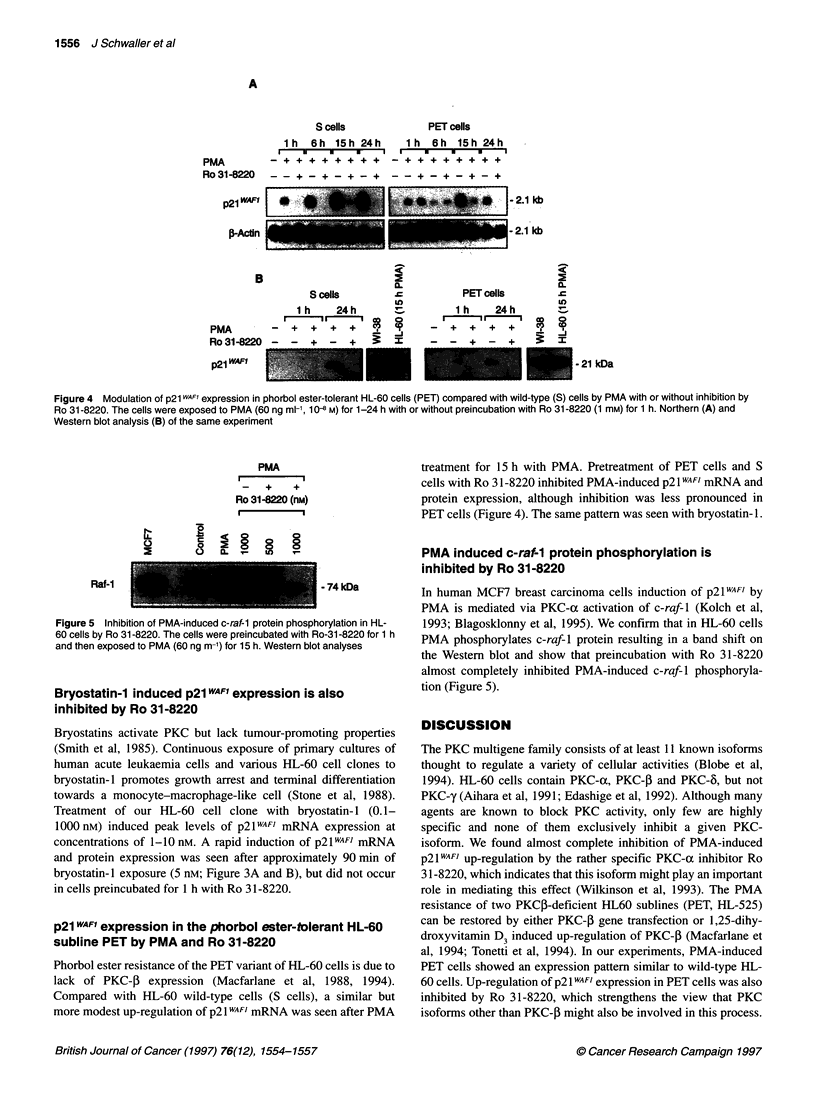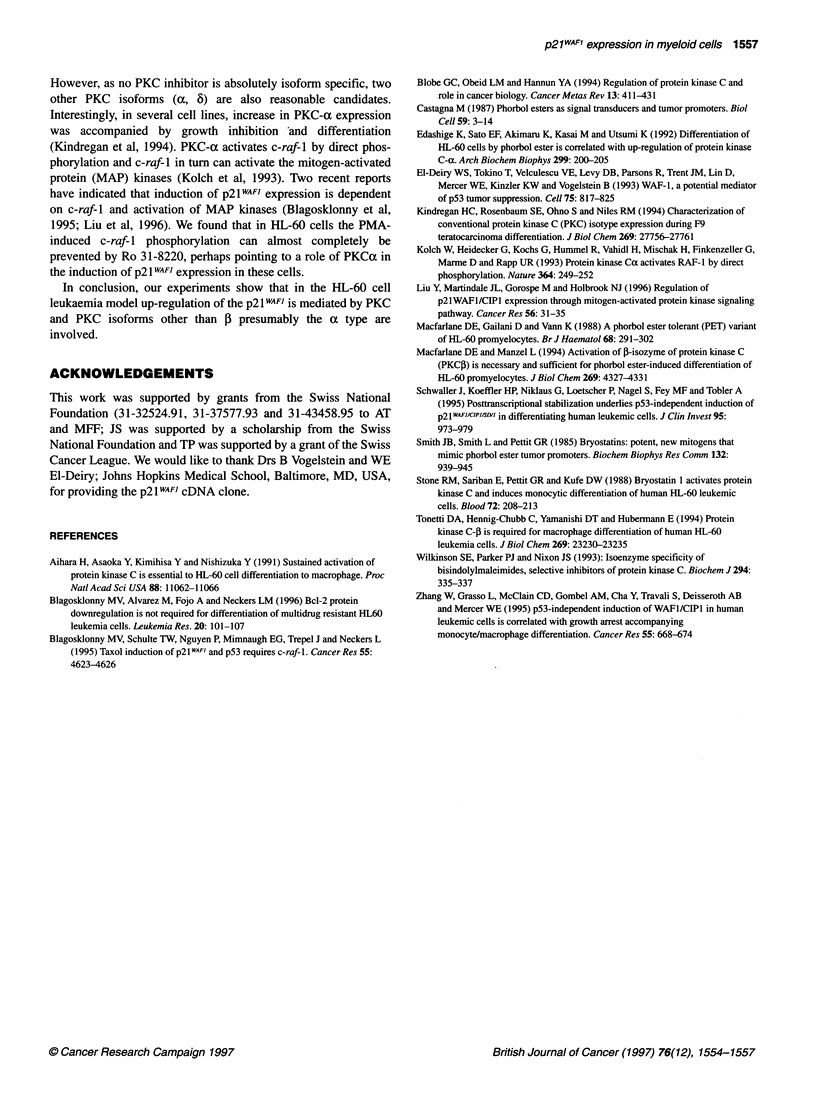# Up-regulation of p21WAF1 expression in myeloid cells is activated by the protein kinase C pathway.

**DOI:** 10.1038/bjc.1997.595

**Published:** 1997

**Authors:** J. Schwaller, U. R. Peters, T. Pabst, G. Niklaus, D. E. Macfarlane, M. F. Fey, A. Tobler

**Affiliations:** The Department of Clinical Research, University of Berne, Switzerland.

## Abstract

**Images:**


British Journal of Cancer (1997) 76(12), 1554–1557
© 1997 Cancer Research Campaign

Short communication

### Up-regulation of p21$^{WAF1}$ expression in myeloid cells is activated by the protein kinase C pathway

J Schwaller[1], UR Peters[1,2], Th Pabst[1], G Niklaus[1], DE Macfarlane[4], MF Fey[1,2] and A Tobler[1,3]

[1]The Department of Clinical Research, [2]Institute of Medical Oncology, [3]Central Haematology Laboratory, University of Berne, Berne, Switzerland; and [4]Department of Medicine, Veterans Administration Hospital and University of Iowa, IA, USA

**Summary** Phorbol-12-myristate-13-acetate (PMA) induces p21$^{WAF-1}$ expression in human myeloid leukaemic HL-60 cells. We show that this induction is specifically mediated by protein kinase C (PKC). In addition, the PKC inhibitor Ro 31-8220 with predominant PKC-α isoform specificity almost completely inhibited PMA-induced up-regulation of p21$^{WAF1}$ in HL-60 cells as well as in the myelomonocytic leukaemic U937 cells. Pretreatment of HL-60 cells with Ro 31-8220 also inhibited PMA-induced activation of c-*raf*-1, a known PKC α target. In the *p*horbol *e*ster-*t*olerant HL-60 subline (PET) with PKC-β isoform deficiency PMA or bryostatin-1 induced p21$^{WAF1}$ expression, but to a lesser extent than in wild-type HL-60 cells. In PET cells, Ro 31-8220 also inhibited PMA and bryostatin-1-induced up-regulation of p21$^{WAF1}$ expression. Our findings indicate that at least in HL-60 cells up-regulation of p21$^{WAF-1}$ is specifically activated by PKC. We suggest that PKC isoforms other than β, presumably the PKC-α isoform, are involved in this process.

Cancer is characterized by profound alterations both in cell cycle control and in cellular differentiation. Such mechanisms can be studied with the help of cell line models, for example leukaemic cell lines in which both proliferation and differentiation programmes may be analysed. Phorbol ester-induced differentiation of human leukaemic myeloid HL-60 cells towards monocyte-like cells is associated with growth arrest in the $G_1$ phase of the cell cycle. The CKI p21$^{WAF1}$ (*w*ild-type p53-*a*ctivated *f*ragment-1) is a mediator of $G_1$ cycle arrest induced by wild-type p53 protein (El-Deiry et al, 1993), and in human leukaemia p21$^{WAF1}$ expression may be up-regulated independently from p53 (Schwaller et al, 1995; Zhang et al, 1995; Blagosklonny et al, 1996).

Phorbol esters are potent activators of protein kinase C (PKC), a family of serine–threonine protein kinases that act as a central mediators of signal transduction pathways (Castagna, 1987). In HL-60 cells concordant expression pattern of PKC-α and -β isoenzymes is seen during phorbol ester-induced monocytic differentiation (Aihara et al, 1991; Edashige et al, 1992). To examine the role of PKC in up-regulating p21$^{WAF1}$ expression, we modulated p21$^{WAF1}$ expression by several PKC activators and inhibitors. We also investigated the expression of c-*raf*-1, a protein serine–threonine kinase required for p21$^{WAF1}$ induction and up-stream regulation of mitogen-activated protein (MAP) kinase (Blagosklonny et al, 1995).

### MATERIAL AND METHODS

### Reagents

PMA, 4β-phorbol 12,13-didecanoate, 4α-phorbol-12-13-didecanoate and mezerein (all from Sigma Chemical, St Louis, MO, USA), were dissolved in acetone to a stock solution of 1 mM and stored at –20°C. Ro 31-8220 ('compound 3"; provided by Dr G Lawton, Roche Research Centre, Welwyn Garden City, Herts, UK), and bryostatin-1 (provided by Dr GR Pettit, Cancer Research Institute, Tempe, AZ, USA) were dissolved in dimethylsulphoxide (DMSO; Merck Chemical, Darmstadt, Germany) to stock solutions of 1 mM.

*Received 6 September 1996*
*Revised 23 May 1997*
*Accepted 3 June 1997*

*Correspondence to:* A Tobler, Central Haematology Laboratory, University of Berne, Inselspital, CH-3010 Berne, Switzerland

### Cell cultures

HL-60-cells (American Type Tissue Culture Collection, ATCC, Rockville, MD, USA), S-cells (HL-60, wild-type) and PET-cells (*p*horbol-*e*ster *t*olerant HL-60) provided by Dr DE Macfarlane, University of lowa, IA, USA, were all cultured at low numbers of passages (< 35) in McCoy's supplemented with 10% heat-inactivated fetal bovine serum in 5% carbon dioxide at 37°C. Normal diploid lung fibroblasts (WI-38; ATCC), expressing high levels of p21$^{WAF1}$, and a human breast cancer cell line MCF7 (provided by Dr A Ziemiecki, Department of Clinical Research, University of Berne, Switzerland), expressing high levels of c-*raf*-1, served as positive controls (Blagosklonny et al, 1995; Schwaller et al, 1995).

### Northern blot analysis

RNA extraction and Northern blotting as well as hybridization were performed as described previously (Schwaller et al, 1995). Human cDNA probes were: p21$^{WAF1}$ (2.1 kb; *Bam*HI-*Hin*dIII) from pCEP (El-Deiry et al, 1993), and β-actin (0.7 kb; *Eco*RI-*Bam*HI, from pHF-A-3'UTR (Schwaller et al, 1995).

### Western blot analysis

Total cellular protein was extracted and Western blotted as described (Schwaller et al, 1995). A polyclonal rabbit anti-human p21$^{WAF1}$ specific antibody (C-19; Santa Cruz, Santa Cruz, CA,

**Figure 1** Effect of different phorbol derivatives on p21 *WAF1* expression in HL-60 cells. The cells were treated with 4β-phorbol 12,13-didecanoate (4β-phorbol), 4α-phorbol 12-13-didecanoate (4α-phorbol), mezerein and phorbol-12-myristate-13-acetate (PMA) as indicated. Northern blot (**A**) and Western blot (**B**) analyses of the same experiment. The same results were obtained in three independent experiments

**Figure 2** (**A**) Inhibition of PMA-induced p21 *WAF1* mRNA expression in HL-60 cells by the PKC inhibitor Ro 31-8220. Cells were preincubated for 1 h with Ro 31-8220 and then exposed to PMA (60 ng ml⁻¹, $10^{-8}$ M) for 15 h. Northern blot analysis. (**B**) Inhibition of PMA-induced p21 *WAF1* protein expression in HL-60 cells and U937 cells by Ro 31-8220. Western blot analysis

**Figure 3** Modulation of p21 *WAF1* expression of HL-60 cells by bryostatin-1. Cells were exposed to bryostatin-1 (Bryo; $5 \times 10^{-9}$ M) for 1–6 h with or without preincubation with Ro 31-8220 (1 mM) for 1 h. Northern blot (**A**) and Western blot analyses (**B**) of the same experiment

USA) and a polyclonal anti-c-*raf*-1 antibody (Santa Cruz) were used at dilutions of 1:250 and 1:750 respectively.

### RESULTS

### Induction of p21 *WAF1* expression in HL-60 cells by phorbol ester derivatives

PMA, 4β-phorbol 12,13-didecanoate and mezerein are biologically active diterpenes that bind to and activate PKC. After treatment of the cells with these agents p21 *WAF1* mRNA and protein expression was up-regulated (Figure 1). Mezerein was the most potent inducer followed by PMA and 4β-phorbol 12,13-didecanoate. To distinguish between PKC-specific receptor-mediated and non-specific effects, HL-60 cells were treated with 4α-phorbol-12-13-didecanoate (10–1500 nM). This agent is structurally closely related to the tumour promoting diterpenes but displays very low affinity for PKC (Castagna, 1987). No increase of p21 *WAF1* expression was seen. Thus, in HL-60 cells induction of p21 *WAF1* expression by PMA is mediated by activation of PKC.

### Dose-dependent inhibition of PMA-induced p21 *WAF1* expression by the PKC inhibitor Ro 31-8220

In contrast to commonly used PKC inhibitors, for example staurosporine, Ro 31-8220 is less potent but more selective (Wilkinson et al, 1993). Preincubation with Ro 31-8220 (500–1000 nM) for 1 h almost completely inhibited PMA-induced p21 *WAF1* mRNA and protein expression (Figure 2A and B) in HL-60 cells and prevented their differentiation as shown by lack of both esterase staining and adherence to plastic. Cell viability remained > 90%. The same experiments were performed with the myelomonocytic U937 cells. Similar to HL-60 cells, Ro 31-8220 completely inhibited the PMA-induced protein expression of p21 *WAF1* (Figure 2B).

© Cancer Research Campaign 1997

**Figure 4** Modulation of p21$^{WAF1}$ expression in phorbol ester-tolerant HL-60 cells (PET) compared with wild-type (S) cells by PMA with or without inhibition by Ro 31-8220. The cells were exposed to PMA (60 ng ml$^{-1}$, $10^{-8}$ M) for 1–24 h with or without preincubation with Ro 31-8220 (1 mM) for 1 h. Northern (**A**) and Western blot analysis (**B**) of the same experiment

**Figure 5** Inhibition of PMA-induced c-*raf*-1 protein phosphorylation in HL-60 cells by Ro 31-8220. The cells were preincubated with Ro-31-8220 for 1 h and then exposed to PMA (60 ng m$^{-1}$) for 15 h. Western blot analyses

### Bryostatin-1 induced p21$^{WAF1}$ expression is also inhibited by Ro 31-8220

Bryostatins activate PKC but lack tumour-promoting properties (Smith et al, 1985). Continuous exposure of primary cultures of human acute leukaemia cells and various HL-60 cell clones to bryostatin-1 promotes growth arrest and terminal differentiation towards a monocyte–macrophage-like cell (Stone et al, 1988). Treatment of our HL-60 cell clone with bryostatin-1 (0.1–1000 nM) induced peak levels of p21$^{WAF1}$ mRNA expression at concentrations of 1–10 nM. A rapid induction of p21$^{WAF1}$ mRNA and protein expression was seen after approximately 90 min of bryostatin-1 exposure (5 nM; Figure 3A and B), but did not occur in cells preincubated for 1 h with Ro 31-8220.

### p21$^{WAF1}$ expression in the *p*horbol *e*ster-*t*olerant HL-60 subline PET by PMA and Ro 31-8220

Phorbol ester resistance of the PET variant of HL-60 cells is due to lack of PKC-β expression (Macfarlane et al, 1988, 1994). Compared with HL-60 wild-type cells (S cells), a similar but more modest up-regulation of p21$^{WAF1}$ mRNA was seen after PMA treatment for 15 h with PMA. Pretreatment of PET cells and S cells with Ro 31-8220 inhibited PMA-induced p21$^{WAF1}$ mRNA and protein expression, although inhibition was less pronounced in PET cells (Figure 4). The same pattern was seen with bryostatin-1.

### PMA induced c-*raf*-1 protein phosphorylation is inhibited by Ro 31-8220

In human MCF7 breast carcinoma cells induction of p21$^{WAF1}$ by PMA is mediated via PKC-α activation of c-*raf*-1 (Kolch et al, 1993; Blagosklonny et al, 1995). We confirm that in HL-60 cells PMA phosphorylates c-*raf*-1 protein resulting in a band shift on the Western blot and show that preincubation with Ro 31-8220 almost completely inhibited PMA-induced c-*raf*-1 phosphorylation (Figure 5).

### DISCUSSION

The PKC multigene family consists of at least 11 known isoforms thought to regulate a variety of cellular activities (Blobe et al, 1994). HL-60 cells contain PKC-α, PKC-β and PKC-δ, but not PKC-γ (Aihara et al, 1991; Edashige et al, 1992). Although many agents are known to block PKC activity, only few are highly specific and none of them exclusively inhibit a given PKC-isoform. We found almost complete inhibition of PMA-induced p21$^{WAF1}$ up-regulation by the rather specific PKC-α inhibitor Ro 31-8220, which indicates that this isoform might play an important role in mediating this effect (Wilkinson et al, 1993). The PMA resistance of two PKCβ-deficient HL60 sublines (PET, HL-525) can be restored by either PKC-β gene transfection or 1,25-dihydroxyvitamin $D_3$ induced up-regulation of PKC-β (Macfarlane et al, 1994; Tonetti et al, 1994). In our experiments, PMA-induced PET cells showed an expression pattern similar to wild-type HL-60 cells. Up-regulation of p21$^{WAF1}$ expression in PET cells was also inhibited by Ro 31-8220, which strengthens the view that PKC isoforms other than PKC-β might also be involved in this process.

© Cancer Research Campaign 1997

However, as no PKC inhibitor is absolutely isoform specific, two other PKC isoforms (α, δ) are also reasonable candidates. Interestingly, in several cell lines, increase in PKC-α expression was accompanied by growth inhibition and differentiation (Kindregan et al, 1994). PKC-α activates c-*raf*-1 by direct phosphorylation and c-*raf*-1 in turn can activate the mitogen-activated protein (MAP) kinases (Kolch et al, 1993). Two recent reports have indicated that induction of p21[WAF1] expression is dependent on c-*raf*-1 and activation of MAP kinases (Blagosklonny et al, 1995; Liu et al, 1996). We found that in HL-60 cells the PMA-induced c-*raf*-1 phosphorylation can almost completely be prevented by Ro 31-8220, perhaps pointing to a role of PKCα in the induction of p21[WAF1] expression in these cells.

In conclusion, our experiments show that in the HL-60 cell leukaemia model up-regulation of the p21[WAF1] is mediated by PKC and PKC isoforms other than β presumably the α type are involved.

### ACKNOWLEDGEMENTS

This work was supported by grants from the Swiss National Foundation (31-32524.91, 31-37577.93 and 31-43458.95 to AT and MFF; JS was supported by a scholarship from the Swiss National Foundation and TP was supported by a grant of the Swiss Cancer League. We would like to thank Drs B Vogelstein and WE El-Deiry; Johns Hopkins Medical School, Baltimore, MD, USA, for providing the p21[WAF1] cDNA clone.

### REFERENCES

Aihara H, Asaoka Y, Kimihisa Y and Nishizuka Y (1991) Sustained activation of protein kinase C is essential to HL-60 cell differentiation to macrophage. *Proc Natl Acad Sci USA* **88**: 11062–11066

Blagosklonny MV, Alvarez M, Fojo A and Neckers LM (1996) Bcl-2 protein downregulation is not required for differentiation of multidrug resistant HL60 leukemia cells. *Leukemia Res.* **20**: 101–107

Blagosklonny MV, Schulte TW, Nguyen P, Mimnaugh EG, Trepel J and Neckers L (1995) Taxol induction of p21[WAF1] and p53 requires c-*raf*-1. *Cancer Res* **55**: 4623–4626

Blobe GC, Obeid LM and Hannun YA (1994) Regulation of protein kinase C and role in cancer biology. *Cancer Metas Rev* **13**: 411–431

Castagna M (1987) Phorbol esters as signal transducers and tumor promoters. *Biol Cell* **59**: 3–14

Edashige K, Sato EF, Akimaru K, Kasai M and Utsumi K (1992) Differentiation of HL-60 cells by phorbol ester is correlated with up-regulation of protein kinase C-α. *Arch Biochem Biophys* **299**: 200–205

El-Deiry WS, Tokino T, Velculescu VE, Levy DB, Parsons R, Trent JM, Lin D, Mercer WE, Kinzler KW and Vogelstein B (1993) WAF-1, a potential mediator of p53 tumor suppression. *Cell* **75**: 817–825

Kindregan HC, Rosenbaum SE, Ohno S and Niles RM (1994) Characterization of conventional protein kinase C (PKC) isotype expression during F9 teratocarcinoma differentiation. *J Biol Chem* **269**: 27756–27761

Kolch W, Heidecker G, Kochs G, Hummel R, Vahidl H, Mischak H, Finkenzeller G, Marme D and Rapp UR (1993) Protein kinase Cα activates RAF-1 by direct phosphorylation. *Nature* **364**: 249–252

Liu Y, Martindale JL, Gorospe M and Holbrook NJ (1996) Regulation of p21WAF1/CIP1 expression through mitogen-activated protein kinase signaling pathway. *Cancer Res* **56**: 31–35

Macfarlane DE, Gailani D and Vann K (1988) A phorbol ester tolerant (PET) variant of HL-60 promyelocytes. *Br J Haematol* **68**: 291–302

Macfarlane DE and Manzel L (1994) Activation of β-isozyme of protein kinase C (PKCβ) is necessary and sufficient for phorbol ester-induced differentiation of HL-60 promyelocytes. *J Biol Chem* **269**: 4327–4331

Schwaller J, Koeffler HP, Niklaus G, Loetscher P, Nagel S, Fey MF and Tobler A (1995) Posttranscriptional stabilization underlies p53-independent induction of p21[WAF1/CIP1/SDI1] in differentiating human leukemic cells. *J Clin Invest* **95**: 973–979

Smith JB, Smith L and Pettit GR (1985) Bryostatins: potent, new mitogens that mimic phorbol ester tumor promoters. *Biochem Biophys Res Comm* **132**: 939–945

Stone RM, Sariban E, Pettit GR and Kufe DW (1988) Bryostatin 1 activates protein kinase C and induces monocytic differentiation of human HL-60 leukemic cells. *Blood* **72**: 208–213

Tonetti DA, Hennig-Chubb C, Yamanishi DT and Hubermann E (1994) Protein kinase C-β is required for macrophage differentiation of human HL-60 leukemia cells. *J Biol Chem* **269**: 23230–23235

Wilkinson SE, Parker PJ and Nixon JS (1993): Isoenzyme specificity of bisindolylmaleimides, selective inhibitors of protein kinase C. *Biochem J* **294**: 335–337

Zhang W, Grasso L, McClain CD, Gombel AM, Cha Y, Travali S, Deisseroth AB and Mercer WE (1995) p53-independent induction of WAF1/CIP1 in human leukemic cells is correlated with growth arrest accompanying monocyte/macrophage differentiation. *Cancer Res* **55**: 668–674

© Cancer Research Campaign 1997